# Real-World Safety and Early Effectiveness of First-Line Enfortumab Vedotin Plus Pembrolizumab with Routine Dexamethasone Premedication in Advanced Urothelial Carcinoma

**DOI:** 10.3390/cancers18050739

**Published:** 2026-02-25

**Authors:** Takuto Hara, Naoto Wakita, Taisuke Tobe, Hideto Ueki, Yasuyoshi Okamura, Yukari Bando, Kotaro Suzuki, Tomoaki Terakawa, Yoji Hyodo, Akihisa Yao, Koji Chiba, Jun Teishima, Hideaki Miyake

**Affiliations:** Department of Urology, Kobe University Graduate School of Medicine, 7-5-1, Kusunoki-cho, Kobe 650-0017, Japan; complus_duefl@yahoo.co.jp (N.W.); ttobe@med.kobe-u.ac.jp (T.T.); far.e.is.w@gmail.com (H.U.); dfgky008@gmail.com (Y.O.); yukaribando@hotmail.co.jp (Y.B.); pikataro1012@gmail.com (K.S.); daatera0804@yahoo.co.jp (T.T.); yhyodo@med.kobe-u.ac.jp (Y.H.); yaaki1919@gmail.com (A.Y.); kchiba@med.kobe-u.ac.jp (K.C.); teishima@med.kobe-u.ac.jp (J.T.); hmiyake@med.kobe-u.ac.jp (H.M.)

**Keywords:** urothelial carcinoma, enfortumab vedotin, pembrolizumab, real-world data, dexamethasone, EVITA

## Abstract

**Background**: Enfortumab vedotin plus pembrolizumab (EVP) has become a first-line standard for metastatic or unresectable urothelial carcinoma. However, EVP is associated with distinct toxicities, particularly cutaneous adverse events, and prophylactic systemic corticosteroids were not permitted in pivotal trials. Therefore, the safety and early effectiveness of EVP with routine dexamethasone premedication in real-world practice remain unclear. **Methods**: This multicenter retrospective study included consecutive patients with metastatic or unresectable urothelial carcinoma who received first-line EVP at five institutions in Japan between September 2024 and September 2025. All patients received routine intravenous dexamethasone premedication before enfortumab vedotin administration. Safety and early clinical outcomes were evaluated, with exploratory subgroup analyses according to clinical trial eligibility and EVITA criteria. **Results**: Seventy-seven patients were included, with a median age of 75 years, and more than half would have been ineligible for pivotal clinical trials. Cutaneous toxicity was the most frequent adverse event (52.0%), whereas grade ≥3 skin reactions were uncommon (3.9%). With a median follow-up of 6.7 months, the objective response rate was 73.0%. The 6-month progression-free survival and overall survival rates were 73.9% and 78.7%, respectively. Early progression-free survival was generally maintained across subgroups stratified by clinical trial eligibility and EVITA category, with no evidence of excess early progression. **Conclusions**: In this real-world multicenter cohort, first-line EVP with routine dexamethasone premedication was feasible, with manageable toxicity and encouraging early clinical activity, including in patients with comorbidities commonly encountered in routine clinical practice. Longer follow-up and prospective comparative studies are warranted to clarify long-term outcomes and the impact of dexamethasone on efficacy and toxicity.

## Simple Summary

Enfortumab vedotin combined with pembrolizumab has become a new first-line treatment for advanced urothelial carcinoma, but it is frequently associated with skin reactions and other side effects. In pivotal clinical trials, routine use of systemic corticosteroids such as dexamethasone was not permitted, and therefore the safety of this supportive strategy in everyday practice has not been well studied in routine practice. In this multicenter real-world study in Japan, we evaluated the safety and early treatment outcomes of this combination when dexamethasone was routinely administered as a premedication. Many patients were elderly or would not have qualified for clinical trials. Severe skin reactions were uncommon, and early disease control was maintained across risk groups. These findings provide practical evidence that this approach is feasible in routine clinical settings and support further prospective studies to clarify its long-term impact.

## 1. Introduction

Systemic therapy for metastatic or unresectable urothelial carcinoma (UC) has undergone a major transformation in recent years. The emergence of immunotherapy-based combination regimens, particularly those combining an antibody–drug conjugate (ADC) with an immune checkpoint inhibitor (ICI), has fundamentally altered the treatment paradigm. Among these, enfortumab vedotin plus pembrolizumab (EVP) has emerged as a key first-line option for advanced UC and is increasingly regarded as a new standard of care [1].

Unlike platinum-based chemotherapy or ICI monotherapy, with which urologists and medical oncologists have accumulated extensive clinical experience, EVP therapy is associated with a distinct toxicity profile. These toxicities arise from both the Nectin-4–targeted ADC component and the concomitant use of immune checkpoint blockade, resulting in adverse events that are unique to this combination regimen [2,3]. Consequently, dose modifications, schedule adjustments, and specific precautions in patient management are frequently required in routine practice [4]. In this context, expert opinions have advocated for careful patient selection using criteria such as the EVITA framework to identify patients at increased risk of treatment-related toxicity [5].

Among treatment-related adverse events, cutaneous toxicity represents one of the most frequent and clinically significant complications of EVP therapy. Skin reactions can range from mild rash to severe and potentially life-threatening conditions, and fatal cases have been reported [6,7]. As a result, the prevention and management of skin toxicity have become a major concern for clinicians administering EVP therapy. Several strategies have been proposed, including dose reduction or interruption of enfortumab vedotin and the prophylactic use of topical corticosteroids [8,9]. However, an optimal and standardized preventive approach has not yet been established. In addition to cutaneous toxicity, pulmonary adverse events such as interstitial lung disease (ILD) have been reported with both ICIs and certain ADC. Given the potential for overlapping immune-mediated and cytotoxic mechanisms in EVP therapy, pulmonary safety warrants attention in routine clinical practice [10,11].

We previously reported that routine dexamethasone premedication during enfortumab vedotin monotherapy in the late-line treatment setting for metastatic UC was associated with a reduced incidence of skin rash [12]. Nevertheless, in pivotal clinical trials evaluating EVP therapy, prophylactic systemic corticosteroid administration was not permitted, and thus the clinical significance of dexamethasone premedication with respect to treatment efficacy and safety remains unclear [1]. In particular, there is concern that systemic corticosteroids might attenuate antitumor immune responses when used concomitantly with ICIs [13], underscoring the need for real-world evidence.

In routine clinical practice, patients treated with EVP are often older and more medically complex than those enrolled in pivotal trials, and many do not meet standard eligibility or EVITA criteria [1,5]. Because prophylactic corticosteroids were not permitted in these trials, the safety and efficacy of EVP with routine dexamethasone premedication in such populations remain unclear. Assessing outcomes according to clinical trial eligibility and EVITA criteria may therefore help clarify the feasibility and generalizability of this approach. To our knowledge, no prior study has specifically evaluated the safety and early oncologic outcomes of first-line EVP administered with routine systemic corticosteroid premedication in a real-world population enriched with elderly and trial-ineligible patients.

## 2. Methods

This retrospective observational study included patients with metastatic or unresectable urothelial carcinoma who received EVP as first-line systemic therapy at Kobe University Hospital and four affiliated institutions. Consecutive patients treated between September 2024, corresponding to the commercial launch of EVP in Japan, and September 2025 were identified. The data cutoff date for the present analysis was 30 November 2025. Patients were required to have a confirmed diagnosis of metastatic or unresectable urothelial carcinoma and to have initiated EVP as first-line systemic treatment. Patients who received EVP primarily based on patient preference without meeting criteria for metastatic disease or technically unresectable cancer were excluded.

The study protocol was approved by the Institutional Review Board of Kobe University (approval number: B250090). Given the retrospective design, informed consent was obtained using an opt-out approach in accordance with institutional and national ethical guidelines.

Baseline disease assessment was performed within 28 days prior to treatment initiation in all patients. This assessment included contrast-enhanced computed tomography of the chest and abdomen and bone scintigraphy to evaluate metastatic lesions. Target lesions were identified and documented according to the Response Evaluation Criteria in Solid Tumors (RECIST), version 1.1. Tumor responses were investigator-assessed at each participating institution without independent central radiologic review, and imaging assessments during treatment were performed in routine practice at the discretion of the treating physicians (typically every 6–8 weeks, when feasible). Clinical data collected at baseline included medical history, performance status, and laboratory evaluations of organ function. Based on these data, eligibility for pivotal clinical trials [1] was retrospectively determined according to the published inclusion and exclusion criteria, applied as strictly as permitted by the available clinical data. Specifically, key eligibility domains assessed included Eastern Cooperative Oncology Group (ECOG) performance status, adequacy of hematologic, hepatic, and renal function, absence of active autoimmune disease requiring systemic immunosuppression, absence of uncontrolled infection, and the presence or absence of symptomatic central nervous system metastases. EVITA status was retrospectively assessed according to the original EVITA framework [5]. The predefined risk factors included hemoglobin A1c > 8% (or fasting plasma glucose > 150 mg/dL on two consecutive measurements), grade ≥ 2 sensory or motor neuropathy, documented corneal or retinal abnormality, impaired renal function (creatinine clearance or estimated glomerular filtration rate ≤ 45 mL/min), and ECOG performance status ≥ 2. Each factor was evaluated using baseline clinical and laboratory data prior to treatment initiation. Patients were categorized according to the number of fulfilled criteria, and those meeting two or more factors were considered higher risk in exploratory analyses. Ocular abnormalities were defined based on previously documented diagnoses in routine clinical practice; no systematic ophthalmologic screening was performed.

The EVP treatment schedule was based on the regimen used in pivotal clinical trials [1]. Dexamethasone (6.6 mg, intravenous) was administered prior to each enfortumab vedotin infusion as part of routine institutional supportive care. This dose corresponds to the standard antiemetic premedication recommended in the 2023 Japan Society of Clinical Oncology guidelines and was not intended as an immunosuppressive intervention [14]. Dose modifications, treatment delays, or temporary interruptions of enfortumab vedotin and/or pembrolizumab were allowed at the discretion of the treating physician according to clinical judgment.

Safety was evaluated by recording the incidence, type, and severity of treatment-related adverse events, which were graded according to the Common Terminology Criteria for Adverse Events (CTCAE), version 5.0. Interstitial lung disease (ILD) was defined as the development of new interstitial infiltrates on chest computed tomography accompanied by compatible respiratory symptoms, including dyspnea, cough, or hypoxia, in the absence of alternative etiologies. When ILD was suspected, diagnostic evaluation included clinical assessment, laboratory testing, and microbiologic studies as appropriate to exclude infectious pneumonia, heart failure–related pulmonary edema, or tumor progression. Radiologic findings were interpreted with reference to formal radiology reports. In cases of suspected ILD, consultation with respiratory medicine specialists was routinely undertaken as part of standard clinical practice to support diagnostic evaluation and management decisions. All adverse events were recorded from treatment initiation until treatment discontinuation or the data cutoff date and are reported for the entire treatment period.

Particular attention was paid to cutaneous toxicities, peripheral neuropathy, and immune-related adverse events. Clinical effectiveness was assessed using progression-free survival (PFS) and overall survival (OS).

Dose modifications were assessed through cycle 3 to capture early tolerability, particularly cutaneous toxicity, which typically emerges during the initial treatment cycles. This time point was also selected because most patients had reached at least three cycles despite variable follow-up durations, enabling a consistent evaluation across the cohort.

Descriptive statistics were used to summarize patient characteristics, treatment exposure, and adverse events. Time-to-event outcomes were estimated using the Kaplan–Meier method. Exploratory subgroup analyses were performed according to clinical trial eligibility and EVITA criteria status. Owing to the exploratory nature of the study, no formal sample size calculation was performed. All statistical analyses were conducted using EZR (version 1.70) [15].

## 3. Results

Between September 2024 and September 2025, a total of 97 patients with advanced urothelial carcinoma were initially identified. Of these, eight patients were deemed unsuitable for systemic therapy based on clinical judgment at treatment initiation, and twelve patients received systemic treatments other than EVP. After applying these exclusion criteria, 77 patients with metastatic or unresectable urothelial carcinoma who received EVP as first-line systemic therapy were included in the final analysis.

Baseline patient characteristics are summarized in Table 1 and Supplementary Table S1. The median age at treatment initiation was 75 years, and the majority of patients were male. Visceral metastases were present in approximately one third of patients. More than half of the cohort had at least one factor rendering them ineligible for pivotal clinical trials. According to the EVITA framework, a substantial proportion of patients met at least one EVITA factor, while a smaller subset fulfilled two or more criteria, reflecting a real-world population enriched with elderly and comorbid patients; notably, six patients fulfilled both clinical trial ineligibility criteria and two or more EVITA factors.

Treatment exposure and early dose modifications through cycle 3 are summarized in Table 2, and individual treatment trajectories are illustrated in Supplementary Figure S1. Most patients initiated enfortumab vedotin at the standard dose, while a small proportion started at a reduced dose. During the first three cycles, dose reductions, day 8 omissions, or treatment delays occurred in a subset of patients. Discontinuation of pembrolizumab alone without permanent cessation of enfortumab vedotin was not observed. Overall, most patients were able to continue EVP therapy beyond the initial treatment cycles.

The incidence and severity of treatment-related adverse events observed during the entire treatment period are summarized in Table 3. Cutaneous toxicity was the most frequently observed adverse event, with rash occurring in 40 patients (52.0%). Grade ≥ 3 skin reactions were uncommon, observed in 3 patients (3.9%). The median time to rash onset was 0.7 months (range, 0.1–6.9), and systemic corticosteroids were required in only 2 patients (2.6%). No grade 3 or higher peripheral neuropathy was observed during the study period. Interstitial lung disease was observed in 13 patients (16.9%), including 8 patients (10.4%) with grade 3 or higher events. Systemic corticosteroid therapy was administered in 11 patients (84.6%). Treatment interruption of both enfortumab vedotin and pembrolizumab occurred in 12 patients; one patient subsequently resumed enfortumab vedotin after clinical improvement. No ILD-related deaths were observed during the study period. Importantly, clinical and/or radiologic improvement was documented in all patients during follow-up.

Tumor response was evaluated according to RECIST criteria. Among the 77 patients included in the study, tumor response was evaluable in 74 patients. An objective response was achieved in 54 patients, including 13 complete responses and 41 partial responses, yielding an objective response rate of 73.0% among evaluable patients. Stable disease was observed in 11 patients, resulting in a disease control rate of 87.8%. Three patients were not evaluable for tumor response.

**Figure 1:**
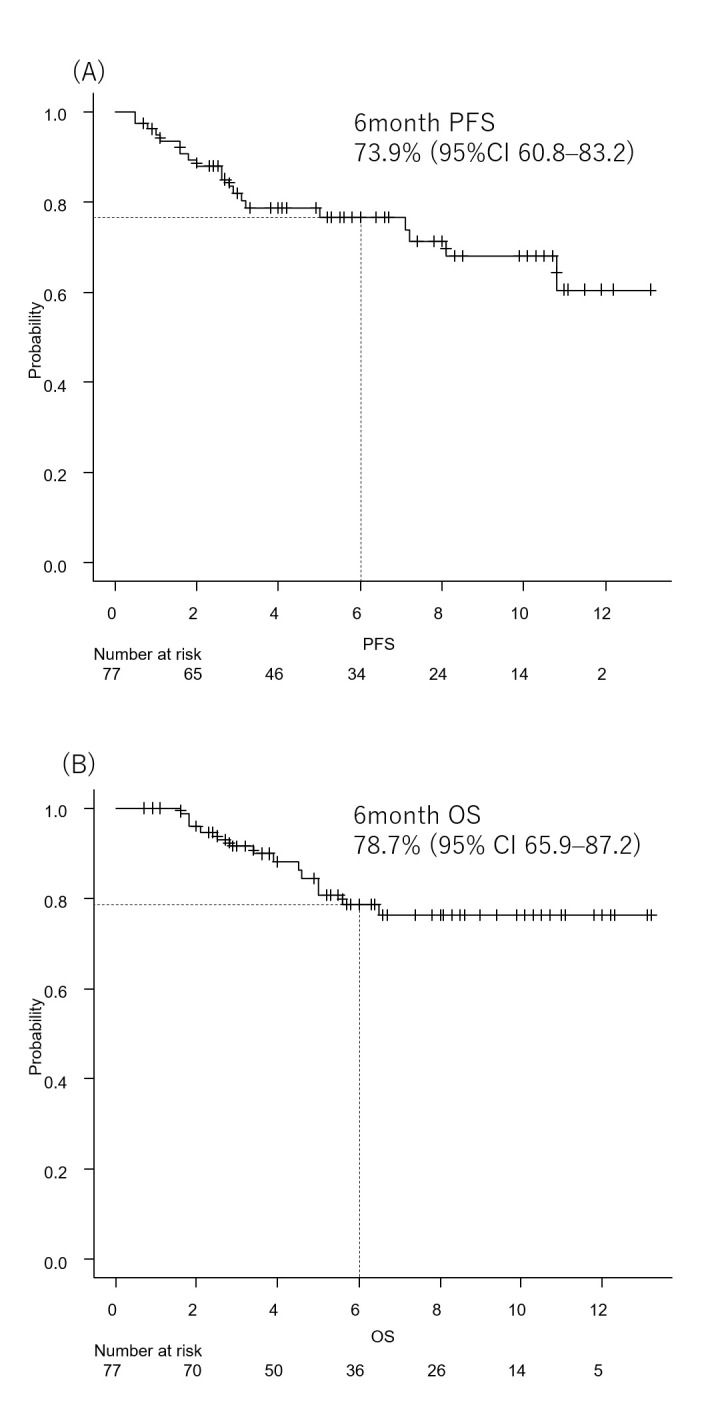
Kaplan–Meier curves in the overall cohort. (**A**) Progression-free survival. (**B**) Overall survival.

With a median follow-up of 6.7 months, PFS and OS outcomes were evaluated. Kaplan–Meier curves for PFS and OS are shown in Figure 1A and Figure 1B, respectively. The 6-month PFS rate was 73.9% (95% confidence interval [CI], 60.8–83.2), and the 6-month OS rate was 78.7% (95% CI, 65.9–87.2). At the time of data cutoff, disease progression had occurred in 20 patients, and 14 deaths were recorded. Median PFS and OS had not yet been reached.

**Figure 2:**
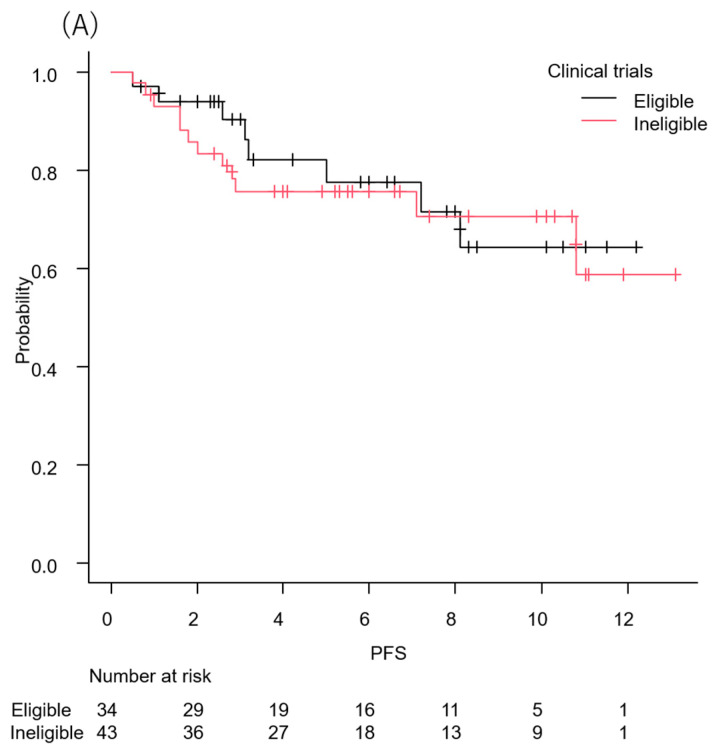

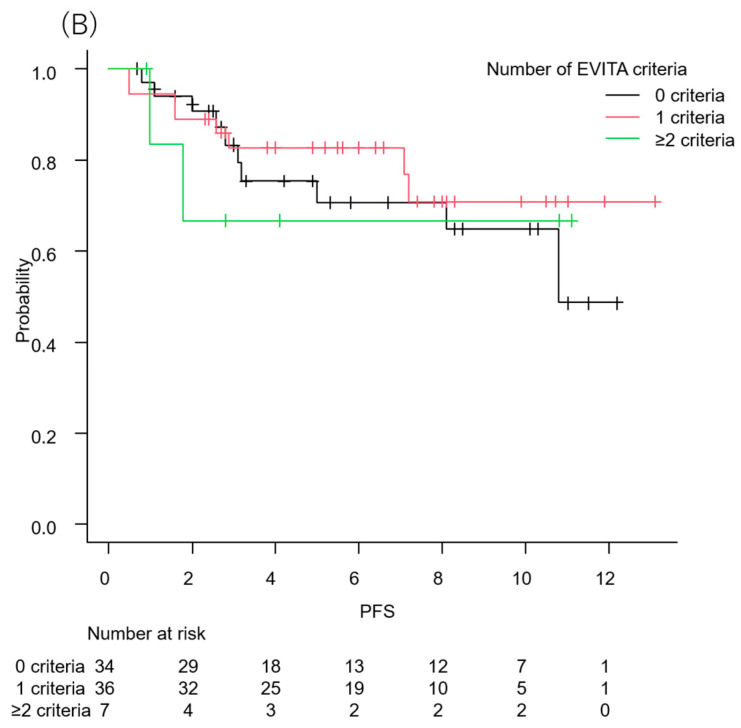
Kaplan–Meier curves for progression-free survival in subgroup analyses. (**A**) Stratified by eligibility for EV-302 trials. (**B**) Stratified by EVITA category.

Exploratory analyses stratified by clinical trial eligibility and EVITA category showed no significant differences in early progression-free survival. Kaplan–Meier analyses demonstrated that progression-free survival was generally maintained during the early treatment period across subgroups, with no evidence of excess early progression events (Figure 2A,B). Objective response rates were also broadly comparable across these subgroups (Supplementary Tables S2 and S3).

The incidence and severity of treatment-related adverse events were also broadly comparable across these subgroups, with no apparent increase in severe toxicities among patients classified as EVITA-positive or ineligible for pivotal clinical trials (Supplementary Tables S4 and S5).

## 4. Discussion

In this multicenter real-world study, we evaluated the feasibility, safety, and early clinical outcomes of first-line enfortumab vedotin plus pembrolizumab administered with routine dexamethasone premedication in patients with metastatic or unresectable urothelial carcinoma, with a specific focus on patient risk profiles defined by clinical trial eligibility and EVITA criteria. To our knowledge, this represents the first real-world analysis to simultaneously examine EVP therapy with prophylactic systemic corticosteroid use across different risk strata in routine clinical practice. Our cohort included a substantial proportion of elderly patients and individuals with comorbidities or clinical features that would have rendered them ineligible for pivotal clinical trials, thereby reflecting everyday clinical settings [1]. Despite these characteristics, EVP therapy with dexamethasone premedication was generally well tolerated, and early disease control appeared to be maintained.

The observed objective response rate and 6-month PFS and OS rates in the present study are broadly consistent with those reported in pivotal clinical trials of EVP therapy [1,16], as well as with outcomes described in recent real-world reports [17,18], although direct comparisons should be avoided given differences in study design, patient characteristics, and follow-up duration. Importantly, no excess of early disease progression was observed, and median PFS and OS had not yet been reached at the time of analysis; therefore, these survival data should be considered preliminary given the relatively short follow-up duration. These findings suggest that effective early disease control with EVP therapy may be achievable even in real-world populations that extend beyond the strict eligibility criteria of clinical trials.

Treatment-related adverse events in our cohort were generally manageable. Cutaneous toxicity was the most frequently observed adverse event, consistent with prior EVP trials [1,16,19]. In EV-103, grade ≥ 3 rash occurred in 17% of patients, and in the global EV-302 study, grade ≥ 3 skin reactions were reported in 15.5% [1,19]. In a pan-Asian EV-302 subgroup [16], grade ≥ 3 maculopapular rash was observed in 11.7%. In contrast, grade ≥ 3 rash occurred in 3.9% of patients in our cohort during the early treatment period. Although cross-study comparisons should be interpreted cautiously due to differences in adverse event definitions and follow-up duration, severe cutaneous toxicity did not appear higher in this Japanese real-world population. The median time to onset of rash in our cohort was 0.7 months, which is consistent with the early onset reported in prior EVP trials [1]. However, the absence of a comparator group without corticosteroid premedication precludes conclusions regarding any mitigating effect of dexamethasone. These findings therefore support the tolerability of EVP with routine dexamethasone in routine practice but do not establish a causal relationship between corticosteroid use and reduced toxicity.

Immune-related adverse events occurred at rates comparable to prior studies [1,16,19], and systemic corticosteroid treatment was required in a limited number of patients, most commonly for the management of interstitial lung disease. Immune checkpoint inhibitor–related pneumonitis has been reported in approximately 3–5% of patients receiving ICI monotherapy in large meta-analyses [10]. Recent pooled safety analyses further indicate that pneumonitis risk may increase with combination regimens compared with ICI monotherapy, and antibody–drug conjugate–associated interstitial lung disease has also been reported across solid tumors [11], supporting the concept that combined immune activation and cytotoxic mechanisms may contribute to pulmonary toxicity [20]. Mechanistically, immune checkpoint blockade may lower the threshold for inflammatory lung injury, while ADC-related epithelial damage could provide a substrate for amplified immune-mediated responses. This dual-hit hypothesis has been proposed as a potential explanation for increased pulmonary toxicity observed with certain combination regimens [21]. In addition, several analyses have suggested that the incidence of pneumonitis may be higher in Asian populations than in non-Asian cohorts [22]. Although a numerically higher incidence of pneumonitis has been reported in pan-Asian populations treated with EVP therapy [16], the frequency observed in our cohort during the early treatment period was not markedly increased.

A key feature of this study is the routine use of dexamethasone premedication prior to enfortumab vedotin administration, consistent with national antiemetic guideline recommendations in Japan [14], as well as international MASCC/ESMO guidelines, which endorse short-course corticosteroids as standard prophylaxis for chemotherapy-induced nausea and vomiting [23]. In pivotal clinical trials of EVP therapy, prophylactic systemic corticosteroids were not permitted [1], and therefore the impact of such premedication on safety and efficacy has remained unclear. In the present cohort, severe cutaneous toxicity was infrequent during the early treatment period; however, the absence of a comparator group without corticosteroid premedication precludes conclusions regarding any mitigating effect of dexamethasone. From a mechanistic perspective, EVP-related skin toxicity is thought to be mediated primarily by on-target effects of enfortumab vedotin via Nectin-4 expression in normal keratinocytes and the cytotoxic activity of the MMAE payload [6,24]. Histopathologic analyses have demonstrated keratinocyte apoptosis accompanied by inflammatory cell infiltration, suggesting that direct cytotoxic injury may be followed by secondary immune-mediated amplification [25,26]. It is therefore biologically plausible that short-term corticosteroid exposure may attenuate inflammatory amplification rather than directly prevent keratinocyte injury. Dexamethasone use in this study reflected routine supportive care practice, in which corticosteroids are widely administered as antiemetics rather than as immunomodulatory interventions [14,23]. The cumulative dexamethasone exposure through cycle 3 was limited, with a median of six administrations (corresponding to approximately 40 mg in total), suggesting short-term antiemetic use rather than sustained systemic immunosuppression.

Concerns have been raised that systemic corticosteroids might attenuate antitumor immune responses when used concomitantly with immune checkpoint inhibitors [13,27]. Although early disease control and short-term survival outcomes were numerically comparable to those reported in pivotal trials, these findings should be interpreted as demonstrating clinical feasibility in routine practice rather than preservation of antitumor immune activity. Theoretical concerns have also been raised regarding potential blunting of immune priming when dexamethasone is administered at treatment initiation [28]. A potential impact on long-term oncologic outcomes cannot be excluded. Prior evidence suggests that corticosteroids administered for non–cancer-related indications may not substantially impair short-term immune checkpoint inhibitor efficacy [13,29,30]. Prospective comparative studies with longer follow-up are warranted.

As antibody–drug conjugates [31] and molecularly targeted agents [32] continue to evolve in urothelial carcinoma, emerging translational data have revealed complex resistance mechanisms and dynamic tumor microenvironment remodeling, while broader multi-omics integration strategies further emphasize the shift toward increasingly personalized treatment paradigms in oncology [33]. These developments underscore the importance of aligning oncologic efficacy with careful toxicity management in routine clinical practice.

The EVITA framework [5] was developed to identify patients at increased risk of toxicity during EVP therapy. However, its predictive accuracy has not yet been fully validated in large prospective cohorts. In our cohort, only a small subset of patients fulfilled two or more EVITA criteria, and most of these patients also exhibited clinical characteristics associated with ineligibility for pivotal clinical trials. Despite these high-risk features, early progression-free survival appeared to be preserved, with no evidence of disproportionate early treatment failure or unacceptable toxicity. These findings are consistent with prior Japanese real-world analyses conducted in the pre-EVP era [34,35], which suggested that EVITA-positive patients represent a minority of cases and largely overlap with clinically frail populations underrepresented in clinical trials. Subsequent validation-oriented analyses, including studies evaluating modified EVITA criteria, have similarly suggested modest discriminatory performance, particularly for toxicity risk stratification, while their impact on survival prediction appears limited [36]. International real-world data likewise indicate that EVITA-positive status frequently overlaps with pivotal trial ineligibility rather than defining a clearly distinct prognostic subgroup. Together, these observations suggest that EVITA should not be viewed as an absolute exclusion criterion, but rather as a tool to support individualized risk assessment and monitoring when EVP therapy is administered in routine practice, and our subgroup analyses should therefore be interpreted as exploratory.

Several limitations of this study should be acknowledged. First, the retrospective and observational design introduces the potential for selection bias and unmeasured confounding. Second, the median follow-up of 6.7 months is appropriate for an early real-world analysis but remains relatively short, and therefore survival outcomes should be regarded as preliminary and subject to change with longer observation. Third, the absence of a control group without dexamethasone premedication limits our ability to directly attribute the observed safety profile to this intervention. Furthermore, although dexamethasone dosing followed national antiemetic guidelines, cumulative exposure was not prospectively standardized. Finally, subgroup analyses based on EVITA criteria and clinical trial eligibility were exploratory in nature and should be interpreted with caution. Given the relatively small sample size in certain subgroups, particularly patients meeting multiple EVITA criteria, these analyses were not powered to detect modest differences in outcomes.

Notwithstanding these limitations, the present findings provide pragmatic insight into the real-world implementation of EVP therapy in routine clinical practice. In contemporary settings, patients treated with EVP frequently extend beyond the strict eligibility criteria of pivotal trials, and supportive strategies such as short-course corticosteroid administration are often incorporated according to institutional practice. Within this context, our data did not demonstrate a clear signal of compromised early disease control or unexpected toxicity during the initial treatment period. Although causal inference cannot be established in the absence of a comparator cohort, these observations suggest that guideline-based dexamethasone premedication may be integrated into first-line EVP treatment without obvious early safety concerns. These results should be regarded as hypothesis-generating and require confirmation in prospective comparative studies with longer follow-up.

## 5. Conclusions

In this multicenter real-world cohort, first-line EVP administered with routine dexamethasone premedication was feasible in patients with metastatic or unresectable urothelial carcinoma, including those with clinical characteristics often underrepresented in pivotal trials. Early safety and short-term disease control outcomes were generally consistent with prior reports; however, the absence of a comparator group without corticosteroid use precludes conclusions regarding the specific impact of dexamethasone on efficacy or toxicity. Given the limited follow-up, these findings should therefore be interpreted as descriptive real-world data supporting feasibility in routine practice. Prospective comparative studies with longer follow-up are warranted to clarify the role of systemic corticosteroid premedication in this setting.

## Figures and Tables

**Table 1:** Baseline patient characteristics.

Characteristics	Total (*n* = *77*)
**Age, years (IQR)**	75 (67–80)
**Sex, *n* (%)**	
Male	61 (79.2)
Female	16 (20.8)
**ECOG performance status, *n* (%)**	
0	27 (35.1)
1	36 (46.8)
2	11 (14.3)
3	3 (3.9)
**Body mass index, kg/m^2^ (IQR)**	21.7 (19.9–23.8)
**Histology, *n* (%)**	
Pure urothelial carcinoma	68 (88.3)
Variant urothelial carcinoma	7 (9.1)
Non-urothelial carcinoma	2 (2.6)
**Primary tumor site, *n* (%)**	
Upper tract urothelial carcinoma	30 (39.0)
Bladder carcinoma	42 (54.5)
Both	5 (6.5)
**Prior radical surgery, *n* (%)**	36 (46.8)
**Visceral metastasis, *n* (%)**	25 (32.5)
Lung metastasis	22 (28.6)
Liver metastasis	6 (7.8)
Bone metastasis	13 (16.9)
**Sum of target lesion diameters, mm (IQR)**	45 (20–74)
**Prior perioperative therapy, *n* (%)**	
Platinum-based neoadjuvant chemotherapy within 1 year	10 (13.0)
Platinum-based adjuvant chemotherapy within 1 year	2 (2.6)
Prior adjuvant nivolumab	6 (7.8)
**EVITA factors, *n* (%)**	
At least one factor	43 (55.8)
Two or more factors	7 (9.1)
**Ineligible for pivotal clinical trials, *n* (%)**	
At least one criterion	44 (57.1)

**Table 2:** Treatment exposure and early dose modifications through cycle 3.

Variable	*n* (%)
**Starting dose of EV on cycle 1 day 1 (mg/kg)**	
1.25	71 (92.2)
1.0	5 (6.5)
0.75	1 (1.3)
**Discontinuation of EV before completion of cycle 3**	
Due to adverse events	3 (3.9)
Due to disease progression	7 (9.1)
Ongoing treatment at data cutoff (cycle 3 not yet completed)	4 (5.2)
**Any EV dose reduction through cycle 3**	9 (11.7)
**Any omission of day 8 dosing through cycle 3**	17 (22.1)
**Any treatment delay through cycle 3**	3 (3.9)
Relative dose intensity of EV through cycle 3, % (IQR) *	93 (80–100)
**Dexamethasone administrations through cycle 3, median (IQR) †**	6 (5–6)
**Pembrolizumab discontinuation alone (EV continued)**	0 (0)

* Relative dose intensity (RDI) was calculated through cycle 3 and is presented as median (interquartile range), excluding patients who were still receiving treatment at the data cutoff. † Dexamethasone (6.6 mg) was administered at each enfortumab vedotin infusion as antiemetic premedication; therefore, the number of administrations corresponded to the number of EV infusions delivered.

**Table 3:** Treatment-related adverse events.

Adverse Event	All Grade, *n* (%)	Grade ≥ 3, *n* (%)	Time to Onset, Months (Range)	Systemic Corticosteroid Use Beyond Routine Premedication, *n* (%)
Rash	40 (52.0)	3 (3.9)	0.7 (0.1–6.9)	2 (2.6)
Dysgeusia	26 (33.8)	0	1.6 (0–5.1)	0
Alopecia	19 (24.7)	0	1.0 (0.6–4.2)	0
Peripheral neuropathy	18 (23.4)	0	2.1 (0.4–8.8)	0
Fatigue	15 (19.5)	1 (1.3)	—	0
Interstitial lung disease	13 (16.9)	8 (10.4)	—	11 (14.3)
Diarrhea	10 (13.0)	3 (3.9)	—	1 (1.3)
Hepatic dysfunction	5 (6.5)	3 (3.9)	—	1 (1.3)
Hyperglycemia	4 (5.2)	1 (1.3)	—	0
Anemia	3 (3.9)	0	—	0
Adrenal insufficiency	2 (2.6)	1 (1.3)	—	2 (2.6)
Thyroid dysfunction	2 (2.6)	0	—	0
Stomatitis	2 (2.6)	0	—	0
Nausea	2 (2.6)	0	—	0
Cholangitis	1 (1.3)	1 (1.3)	—	0
Cerebral infarction	1 (1.3)	1 (1.3)	—	0
Pulmonary embolism	1 (1.3)	1 (1.3)	—	0
Neutropenia	1 (1.3)	1 (1.3)	—	0
Edema	1 (1.3)	0	—	0

Adverse e vents were graded according to CTCAE version 5.0. Time to onset is shown for selected adverse events with sufficient data. Systemic steroid use indicates treatment for management of the adverse event.

## Data Availability

The data presented in this study are available from the corresponding author upon reasonable request.

## Supplementary Materials

The following supporting information can be downloaded at: https://www.mdpi.com/article/10.3390/cancers18050739/s1, Figure S1: Swimmer plot of individual treatment exposure, with color and pattern indicating enfortumab vedotin dose intensity and day 8 omission, and markers indicating key clinical events and subsequent therapies, Table S1: EVITA factors and clinical trial ineligibility criteria, Table S2: Treatment-related adverse events according to clinical trial eligibility, Table S3: Treatment-related adverse events according to clinical trial eligibility, Table S4: Best Overall Response According to Clinical Trial Eligibility, Table S5: Best Overall Response According to EVITA Category.
