# Supplementary material for: Real-World Safety and Early Effectiveness of First-Line Enfortumab Vedotin Plus Pembrolizumab with Routine Dexamethasone Premedication in Advanced Urothelial Carcinoma
Source: Cancers (Basel). 2026 Feb 25;18(5):739. doi: 10.3390/cancers18050739 (PMC12984957; doi:10.3390/cancers18050739)
Supplement: Supplementary file 1 [file cancers-18-00739-s001.zip › supplemental table 1.pdf]

Supplementary Table S1. EVITA factors and clinical trial ineligibility criteria

Variable	n (%)
**EVITA factors**	
At least one factor	43 (55.8)
Two or more factors	7 (9.1)
Renal dysfunction	30 (39.0)
Poor performance status	11 (14.3)
Prior immune checkpoint inhibitor therapy	6 (7.8)
HbA1c $\geq$ 8.0%	3 (3.9)
Pre-existing peripheral neuropathy	1 (1.3)
**Ineligible for pivotal clinical trials**	
At least one criterion	44 (57.1)
Prior systemic therapy	17 (22.1)
Anemia	16 (20.8)
PS-related factors	11 (14.3)
Renal dysfunction	10 (13.0)
Diabetes mellitus-related factors	5 (6.5)
No measurable target lesions	3 (3.9)
Hepatic dysfunction	2 (2.6)
Active double cancer	2 (2.6)
Histological exclusion	2 (2.6)
Brain metastasis	1 (1.3)
