# Supplementary material for: Real-World Safety and Early Effectiveness of First-Line Enfortumab Vedotin Plus Pembrolizumab with Routine Dexamethasone Premedication in Advanced Urothelial Carcinoma
Source: Cancers (Basel). 2026 Feb 25;18(5):739. doi: 10.3390/cancers18050739 (PMC12984957; doi:10.3390/cancers18050739)
Supplement: Supplementary file 1 [file cancers-18-00739-s001.zip › supplemental table 2.pdf]

Supplementary Table S2. Treatment-related adverse events according to clinical trial eligibility

**Adverse event**	**Trial-eligible (n = 33)**		**Trial-ineligible (n = 44)**	
	All grade, n (%)	Grade ≥3, n (%)	All grade, n (%)	Grade ≥3, n (%)
Rash	18 (54.5)	0 (0)	22 (50.0)	3 (6.8)
Dysgeusia	11 (33.3)	0 (0)	15 (34.1)	0 (0)
Alopecia	7 (21.2)	0 (0)	10 (22.7)	0 (0)
Peripheral neuropathy	6 (18.2)	0 (0)	12 (27.3)	0 (0)
Fatigue	4 (12.1)	1 (3.0)	11 (25.0)	0 (0)
Interstitial lung disease	4 (12.1)	2 (6.1)	9 (20.5)	6 (13.6)
Diarrhea	6 (18.2)	2 (6.1)	4 (9.1)	1 (2.3)
Hepatic dysfunction	2 (6.1)	1 (3.0)	3 (6.8)	1 (2.3)
Hyperglycemia	1 (3.0)	1 (3.0)	3 (6.8)	0 (0)
