# Supplementary material for: Real-World Safety and Early Effectiveness of First-Line Enfortumab Vedotin Plus Pembrolizumab with Routine Dexamethasone Premedication in Advanced Urothelial Carcinoma
Source: Cancers (Basel). 2026 Feb 25;18(5):739. doi: 10.3390/cancers18050739 (PMC12984957; doi:10.3390/cancers18050739)
Supplement: Supplementary file 1 [file cancers-18-00739-s001.zip › supplemental table 4.pdf]

Supplementary Table S4. Best Overall Response According to Clinical Trial Eligibility

Best overall response	Eligible for clinical trials (N=33)	Ineligible for clinical trials (N=44)	P value
Complete response (CR)	3 (9.1%)	10 (22.7%)	0.189
Partial response (PR)	22 (66.7%)	19 (43.2%)	
Stable disease (SD)	3 (9.1%)	8 (18.2%)	
Progressive disease (PD)	3 (9.1%)	6 (13.6%)	
Not evaluable	2 (6.1%)	1 (2.3%)	
