# Supplementary material for: Real-World Safety and Early Effectiveness of First-Line Enfortumab Vedotin Plus Pembrolizumab with Routine Dexamethasone Premedication in Advanced Urothelial Carcinoma
Source: Cancers (Basel). 2026 Feb 25;18(5):739. doi: 10.3390/cancers18050739 (PMC12984957; doi:10.3390/cancers18050739)
Supplement: Supplementary file 1 [file cancers-18-00739-s001.zip › supplemental table 5.pdf]

Supplementary Table S5. Best Overall Response According to EVITA Category

**Best overall response**	**EVITA negative (0 criteria) (N=34)**	**EVITA 1 criterion (N=36)**	**EVITA ≥2 criteria (N=7)**	**P value**
Complete response (CR)	6 (17.6%)	5 (13.9%)	2 (28.6%)	0.160
Partial response (PR)	18 (52.9%)	22 (61.1%)	1 (14.3%)	
Stable disease (SD)	4 (11.8%)	4 (11.1%)	3 (42.9%)	
Progressive disease (PD)	3 (8.8%)	5 (13.9%)	1 (14.3%)	
Not evaluable	3 (8.8%)	0	0	
