# Supplementary figures and images for: Real-World Safety and Early Effectiveness of First-Line Enfortumab Vedotin Plus Pembrolizumab with Routine Dexamethasone Premedication in Advanced Urothelial Carcinoma
Source: Cancers (Basel). 2026 Feb 25;18(5):739. doi: 10.3390/cancers18050739 (PMC12984957; doi:10.3390/cancers18050739)

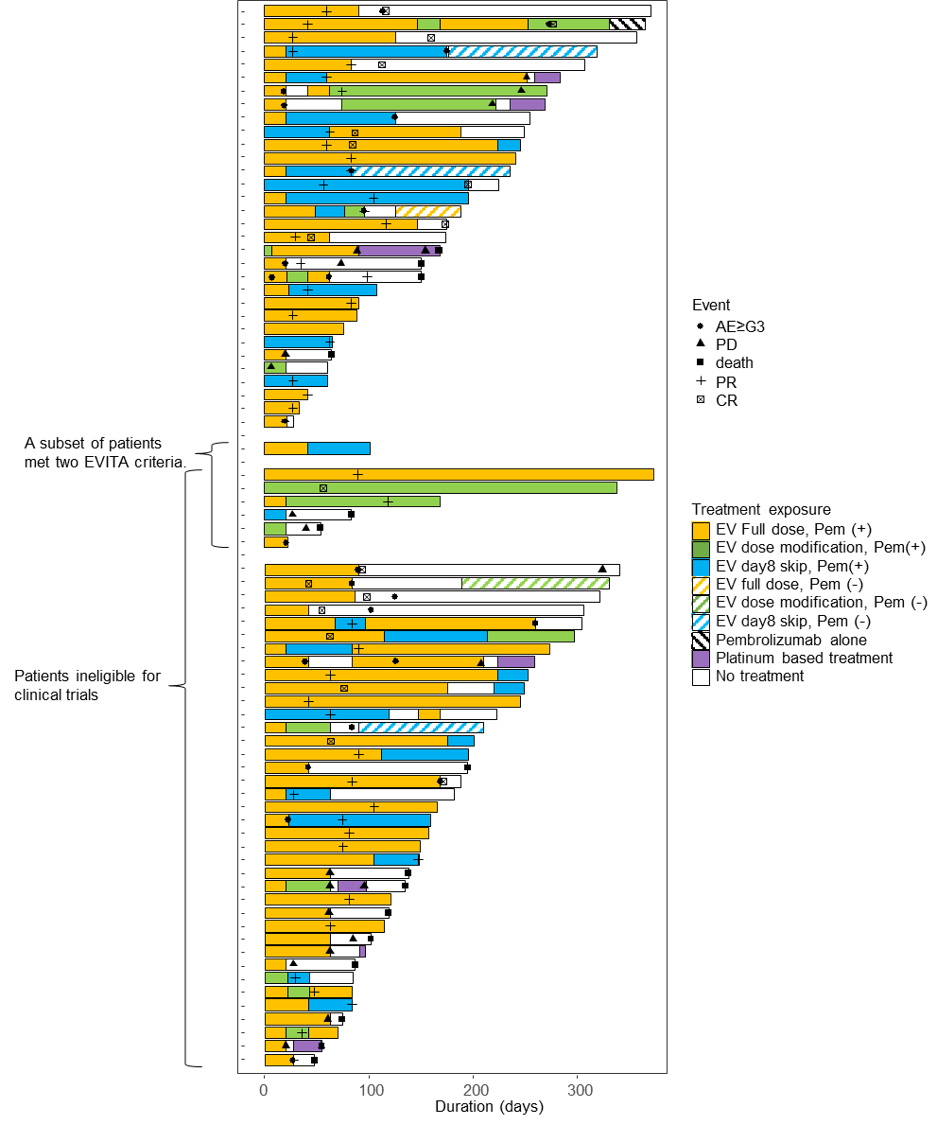

Supplement: Supplementary file 1 [file cancers-18-00739-s001.zip › supplemental figure.png]
